# Anterior meniscus extrusion is associated with anterior tibial osteophyte width in knee osteoarthritis – The Bunkyo Health Study

**DOI:** 10.1016/j.ocarto.2023.100364

**Published:** 2023-04-20

**Authors:** Arepati Adili, Haruka Kaneko, Takako Aoki, Lizu Liu, Yoshifumi Negishi, Jun Tomura, Suguru Wakana, Masahiro Momoeda, Hitoshi Arita, Shinnosuke Hada, Jun Shiozawa, Mitsuaki Kubota, Yuki Someya, Yoshifumi Tamura, Shigeki Aoki, Hirotaka Watada, Ryuzo Kawamori, Takako Negishi-Koga, Yasunori Okada, Muneaki Ishijima

**Affiliations:** a Department of Orthopaedics, Juntendo University Faculty of Medicine, Tokyo, Japan; b Department of Medicine for Orthopaedics and Motor Organ, Juntendo University Graduate School of Medicine, Tokyo, Japan; c Sportology Center, Juntendo University Graduate School of Medicine, Tokyo, Japan; d Department of Pathophysiology for Locomotive Diseases, Juntendo University Graduate School of Medicine, Tokyo, Japan; e Department of Community Medicine and Research for Bone and Joint Diseases, Juntendo University Graduate School of Medicine, Tokyo, Japan; f Department of Metabolism and Endocrinology, Juntendo University Graduate School of Medicine, Tokyo, Japan; g Department of Radiology, Juntendo University Graduate School of Medicine, Tokyo, Japan

**Keywords:** Osteoarthritis, Anterior meniscus extrusion (AME), Medial meniscus extrusion (MME), Anterior tibial osteophyte (ATO), And magnetic resonance imaging (MRI)

## Abstract

**Background:**

In knee osteoarthritis (OA), medial meniscus extrudes both medially and anteriorly. We reported that full-length width of medial tibial osteophyte, which comprises cartilage and bone parts, is directly associated with medial meniscus extrusion in early-stage knee OA and hypothesized that anterior tibial osteophyte (ATO) is also associated with anterior meniscus extrusion (AME). Thus, we aimed to examine their prevalence and relationship.

**Methods:**

Elderly subjects (638 females and 507 males; average 72.9 years old) in the Bunkyo Health Study cohort were enrolled. MRI-detected OA changes were evaluated according to the Whole Organ Magnetic Resonance Imaging Score. ATO was evaluated using the method which can assess both cartilage and bone parts of osteophyte by pseudo-coloring images of proton density-weighted fat-suppressed MRI.

**Results:**

Most subjects showed the Kellgren-Lawrence grade 1/2 of the medial knee OA (88.1%), AME (94.3%, 3.7 ​± ​2.2 ​mm), and ATO (99.6%, 4.2 ​± ​1.5 ​mm). Among the OA changes, AME was most closely associated with full-length width of ATO (multivariable β ​= ​0.877, *p* ​< ​0.001). The area under the receiver operating characteristic curve for determining the presence of AME as evaluated by ATO width was 0.75 (95% confidence interval 0.60–0.84, *p* ​< ​0.001). The odds ratio for the presence of AME as evaluated by ATO width at 2.9 ​mm was 7.16 (4.23–12.15, *p* ​< ​0.001, age, gender, BMI, and K-L adjusted).

**Conclusions:**

AME and ATO were inevitably observed in the elderly subjects and AME was closely associated with full-length width of ATO. Our study provides the first evidence on the close relationship between AME and ATO in knee OA.

## 1 Introduction

Osteoarthritis (OA) of the knee is one of the most common motor organ diseases in middle-aged and older populations [1]. The primary event of knee OA joint was believed to be articular cartilage destruction, which was followed by structural changes of other knee joint components such as meniscus, subchondral bone, ligaments, and synovium [2]. However, clinical research using recently developed diagnostic tools such as magnetic resonance imaging (MRI) has indicated that abnormalities in joint structures other than articular cartilage, especially meniscus and subchondral bone, proceed asymptomatically in many years before the onset of radiographic knee OA and suggested that the changes of non-articular cartilage components such as meniscus act as initiator and/or progression factor for the articular cartilage destruction [3].

Meniscus has important functions including shock absorption, load bearing, lubrication, and stability of knee joint, and thus distributes the load on the articular cartilage of femur and tibia over a large area, contributing to protection of articular cartilage from damage [4]. Medial meniscus is attached to the medial tibial plateau by the menisco-tibial ligament, forming the junctional structures of the meniscus and the tibial plateau, i.e., the anterior and posterior roots of the meniscus [[5], [6], [7]]. Therefore, once root tears occur, the medial meniscus is readily extruded to the medial site, causing medial meniscus extrusion (MME) [8]. However, medial meniscus disposition occurs not only medially (MME) but also anteriorly, and the anterior deviation of the meniscus is called as anterior meniscus extrusion (AME) [9]. In case of changes in position and/or morphology of the meniscus, mechanical force to the articular cartilage is inadequately dispersed and the overall biomechanics of the knee joint are altered, resulting in increased risk for cartilage loss and incidence and progression of knee OA [3,8,[10], [11], [12], [13], [14], [15], [16]]. Actually, MME is known to be an important risk factor for the incidence and progression of knee OA [14,[17], [18], [19]]. Since changes in position of the meniscus are generally related to cartilage loss, semi-quantitative evaluation of MME and AME is included in the scoring system of knee OA, i.e., MRI Osteoarthritis Knee Score (MOAKS) [19]. However, in contrast to MME, no or little information is available for the prevalence of AME and the associations between AME and OA changes in the knee joint.

Osteophyte, a cartilage-capped bony overgrowth in the periosteum arising at the junction between cartilage and bone of OA joints [20], is the most common abnormality among middle-aged populations who had no radiographic knee OA [21]. Osteophyte is formed by following a process similar to endochondral ossification [20,22], and thus is composed of both cartilage and bone components [7]. In our previous study, we have reported that in the early-stage knee OA patients without meniscal lesions or tears, full-length (cartilage part plus bone part) width of osteophyte detected by T2 mapping MRI is coincident with MME [7]. Although this study demonstrated the usefulness of T2 mapping MRI for determination of osteophyte, T2 mapping MRI is not applicable for a cohort study due to the time-consuming method. Thus, we have carried out preliminary study to develop a new method by pseudo-colorization of the conventional MRI images, and obtained the similar correlation between full-length width of osteophyte and MME in elderly populations in a population-based large cohort [23]. However, no studies on anterior tibial osteophyte (ATO) and its relationship with AME have been reported.

In the present study, we examined the prevalence of ATO and AME and explored whether there is any association between AME and ATO width among elderly populations using data from the large-scale cohort study, the Bunkyo Health Study (BHS) [[24], [25], [26]].

## 2 Methods

### 2.1 Subjects of the BHS cohort

The BHS is a prospective cohort study over 10 years to identify risk factors for needing long-term care [[24], [25], [26]]. Elderly subjects between 65 and 84 years of age living in Bunkyo-ku, an urban area in Tokyo, Japan were recruited, and a total of 1630 subjects participated from November 2015 to September 2018. The exclusion criteria of this cohort were the elderly who had implanted pacemakers or defibrillators and had diabetes requiring insulin therapy. This study protocol has been approved by the ethics committee of Juntendo University from November in 2015 (No. 2015078, 2016138, 2016131, and 2017121). The study is being carried out in accordance with the principles outlined in the Declaration of Helsinki. Written informed consent was obtained from all the participants at the orientation meeting. Participants were told that they have the right to withdraw from the trial at any time. Collected data are coded with non-identifying numbers and stored securely in password-protected files. Accessibility to the files is limited to principal investigators. The present study was a cross-sectional study using the baseline data from the BHS.

### 2.2 The radiographic evaluation of knee OA

The radiographic OA severity using the Kellgren-Lawrence (K-L) classification was evaluated based on the weight-bearing antero-posterior radiographs of the tibiofemoral joint for both knees using the bilateral standing extended view and based on the weight-bearing postero-anterior radiographs of the tibiofemoral joint with the knee in 45° of flexion [27]. The tibiofemoral angle (TFA) and medial joint space width (JSW) were evaluated based on the long leg standing radiographs and the weight-bearing antero-posterior radiographs of the lower limbs, respectively.

### 2.3 MRI evaluation

The knee joints of the subjects were analyzed by the 0.3-T MRI system (Hitachi AIRIS Vento; Hitachi Medical Corporation, Tokyo, Japan) with a knee coil. Coronal and sagittal proton density-weighted fat-suppressed (PDFS) and pseudo-colored PDFS (PPDFS) spin-echo sequences (TR/TE ​= ​1500/21.2 msec; in-plane resolution ​= ​0.39 ​× ​0.39 ​mm^2^; section thickness ​= ​3 ​mm) were used for the OA structural changes including MME and osteophyte measurement. The MRI data were evaluated in the tibiofemoral joint, and OA structural changes were scored according to the Whole Organ Magnetic Resonance Imaging Score (WORMS) [28]. Each region of a compartment received its own score, and these scores were added together [7]. Among the sagittal PDFS image sections of the medial tibial compartment, a sagittal section passing through the area of the medial tibial plateau where the central weight-bearing and posterior portion of the femoral condyle contacts was selected in each subject and subjected to pseudo-colorization. In typical sagittal images of the normal knee joint without osteophyte, anterior edge of the tibia was almost rectangular in shape (Supplementary Fig. S1A), and osteophyte showed a spur-like lesion, which is attached to the anterior edge of the rectangle-shaped tibia (Supplementary Fig. S1B). AME and ATO width were measured as the distance between the vertical line drawn at the outer edge of the medial meniscus and the vertical line drawn at border of the tibia, making the rectangle-shaped anterior edge of the tibia appear (Supplementary Fig. S1). AME was grouped to grade with 4° (0–3) according to the MOAKS: grade 0, <2 ​mm; grade 1, 2–2.9 ​mm; grade 2, 3–4.9 ​mm; and grade 3, ≥5 ​mm [19]. Osteophyte widths were evaluated by one of the authors of this manuscript (TA) and another author (AA) evaluated the randomly selected 154 osteophyte widths. The reliability within and between these observers (intraclass correlation coefficient) was 0.86 (95% CI: 0.85–0.88) and 0.98 (95% CI: 0.98–0.99), respectively. Intra-observer reliability was 0.93 (95% CI: 0.88–0.96) for AME and 0.90 (95% CI: 0.83–0.95) for ATO.

### 2.4 Evaluation of osteophytes using PPDFS MRI images

According to our previous study [23], we have developed a new method, i.e., PPDFS MRI method, for measuring osteophytes using by pseudo-coloring the PDFS MRI images, which is a sequence conventionally used for MRI evaluation of the knee. Cartilage part of osteophyte was not detectable by PDFS MRI image. However, the cartilage part could be detected when PDFS MRI image was pseudo-colored by selecting color as Royal that allowed the cartilage to be represented by an intermediate color using ImageJ [29], similar to T2 mapping MRI images [TR/TE ​= ​1000/13.8, 27.6, 41.4, 55.2, 69.0 msec, voxel size ​= ​0.42 ​× ​0.42 ​× ​0.202 ​mm^3^] (Siemens Magnetom Verio; Siemens Healthcare, Erlangen, Germany).

### 2.5 Statistical analysis

The associations between AME and other MRI-detected OA structural alterations including JSW, TFA, cartilage lesion, osteophyte, bone marrow abnormality (BMA), subchondral bone cyst (SBC), subchondral bone attrition (SBA), medial meniscus change, and ATO width were analyzed by univariate linear regression and multivariate linear regression analyses. Correlations between ATO width and AME were examined using Spearman's correlation coefficients. Relationship between ATO width and AME grade were assessed by one-way analysis of variance. Odds ratio for the presence of AME (>0 ​mm) were calculated to evaluate the cut-off score for the ATO width. The area under the curve (AUC), which is analogous to the area determined by the receiver operating characteristic (ROC) curve, was estimated for the discriminative value of the prediction models. The null hypothesis was that the data could not distinguish who is the subject with AME. The criterion for accepting the null hypothesis was an AUC <0.70. The *p* values ​< ​0.05 were considered to be significant. All analyses were performed using the SPSS Statistics 25.0 software program (IBM, Armonk, NY).

## 3 Results

### 3.1 Characteristics of the subjects

Among 1630 subjects of the BHS cohort, 1191 subjects underwent both radiography in the standing position and MRI of the knee joint, and 46 subjects who showed lateral type of knee OA (TFA ​< ​173) were excluded. Thus, the remaining 1145 subjects were included in this study (Table 1). They comprised 683 females (55.7%) and 507 males (44.3%), and age of the subjects was 72.9 years of age on average. Body mass index (BMI) of the subjects was 22.8 ​kg/m^2^ on average. All the 1145 subjects showed radiographic knee OA changes: Most of the subjects (970 subjects, 84.7%) had knee OA with ≥K-L grade 2 on radiography and the remaining 175 subjects (15.3%) had early-stage knee OA with K-L grade 1. The TFA of the subjects was 177.6° on average, and medial JSW was 4.8 ​mm on average on radiography. Not only cartilage pathology but also subchondral changes, meniscus change, and osteophyte formation among the OA-related structural changes evaluated by the WORMS were observed on MRI (Table 1).

**Table 1:** Baseline characteristics of the subjects.

Characteristics	Data
Number of subjects	1145
Gender, *n* (%)	Female, 638 (55.7%); Male, 507 (44.3%)
Age, years	72.9 (5.4)
BMI, kg/cm^2^	22.8 (3.0)
Pain VAS (0–100)	8.6 (16.2)
Radiographic findings	
K-L grades 1/2/3/4 (*n*)	175 (15.3%), 834 (72.8%), 93 (8.1%), 43 (3.8%)
TFA, degree	177.6 (2.5)
Medial JSW, mm	4.8 (1.1)
MRI findings	
WORMS	
Cartilage score (0–30)	2.2 (3.7)
BMA score (0–15)	1.0 (1.9)
SBC score (0–15)	1.7 (2.0)
SBA score (0–15)	4.1 (2.4)
OP score (0–35)	9.6 (9.1)
Meniscus score (0–6)	2.9 (2.4)
AME, mm	3.7 (2.2)
ATO width	
Bone part, mm	2.0 (1.3)
Cartilage and bone parts, mm	4.2 (1.5)

Data are expressed as mean value (standard deviation, if not indicated). BMI, body mass index; VAS, visual analog scale; K-L grade, Kellgren-Lawrence grade; TFA, tibiofemoral angle; JSW, joint space width; WORMS, whole-organ magnetic resonance imaging score; BMA, bone marrow abnormality; SBC, subchondral bone cyst; SBA, subchondral bone attrition; OP, osteophyte; AME, anterior meniscus extrusion; ATO, anterior tibial osteophyte.

### 3.2 Evaluation of AME and ATO by PPDFS MRI

**Fig. 1:**
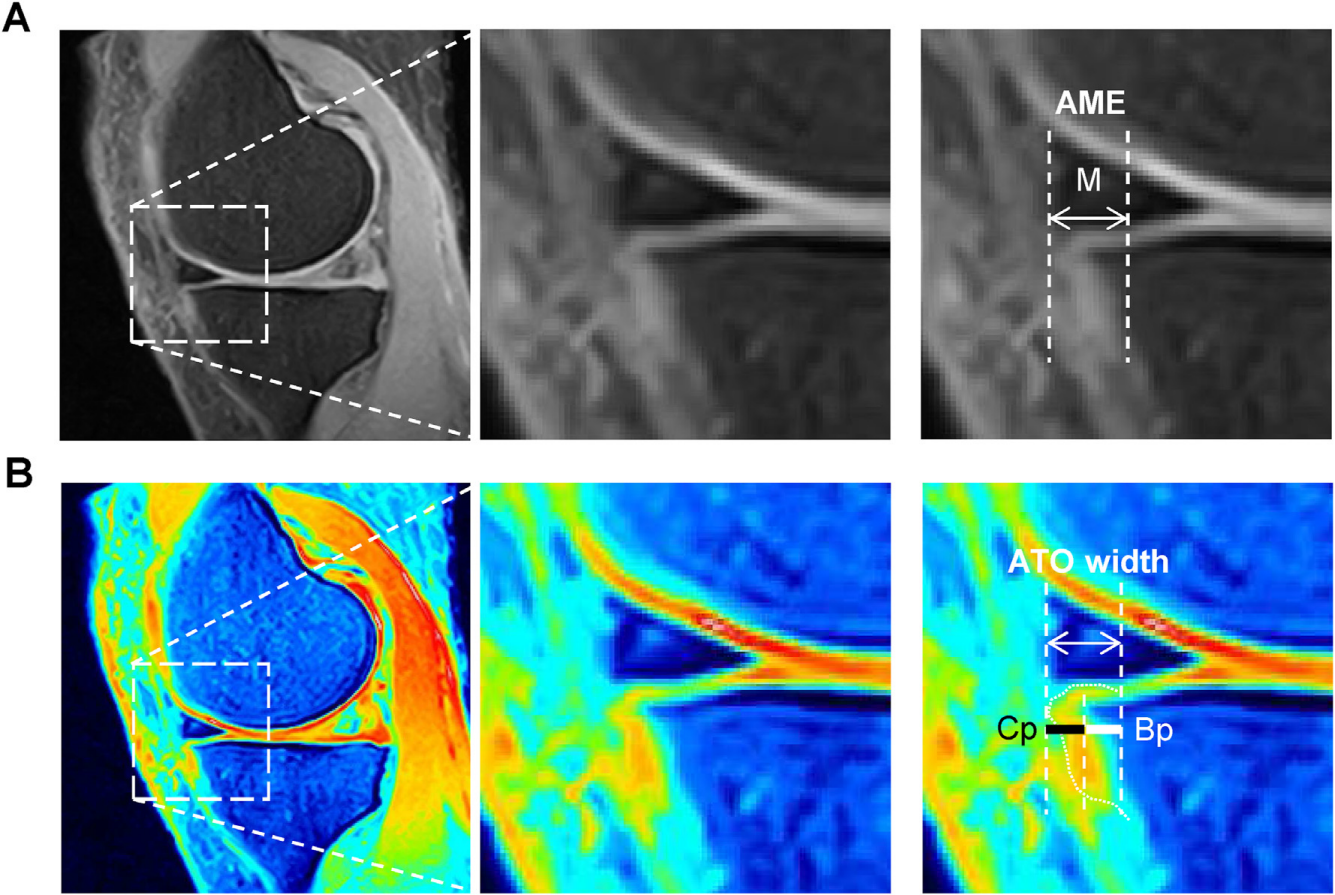
Representative data on the measurement of AME and ATO width by PDFS and PPDFS MRI images of the knee joint, respectively. **(A)**, Measurement of AME on the sagittal PDFS image. AME (white horizontal line with arrows) was measured as the distance between the vertical dotted line drawn at the outer edge of the medial meniscus and the vertical dotted line drawn at border of the tibia, making the rectangle-shaped anterior edge of the tibia appear. **(B)**, Measurement of ATO width using PPDFS image. PPDFS view was prepared by pseudo-coloring the PDFS MRI image as described in the Methods. Vertical dotted lines indicate the anterior edge of the tibia excluding osteophyte, the boundary between the bone part of osteophyte (Bp) (white horizontal line) and cartilage part of osteophyte (Cp) (black horizontal line), and the outer edge of the cartilage part of osteophyte. White horizontal line with arrows indicates full-length (Bp plus Cp) width of ATO. PDFS, proton density-weighted fat-suppressed; PPDFS, pseudo-colored PDFS; M, medial meniscus; AME, anterior meniscus extrusion; ATO, anterior tibial osteophyte.

AME was observed by focusing on the anterior lesion of the knee joint in the sagittal section of PDFS MRI (Fig. 1A). Most of the subjects showed AME and the value of AME in the subjects was 3.7 ​± ​2.2 (mean ​± ​SD) mm (Table 1). Although bone part of ATO was detectable by PDFS MRI, cartilage part of ATO and its border by PDFS MRI were obscure (Fig. 1A). When ATO was analyzed by converting the images of PDFS MRI into PPDFS images, cartilage and bone parts of osteophyte were clearly detected and full-length (cartilage part plus bone part) width of whole ATO was determined (Fig. 1B). The bone part and full-length widths of ATO were 2.0 ​± ​1.3 ​mm and 4.2 ​± ​1.5 ​mm, respectively (Table 1).

### 3.3 Incidence of MRI-detected OA structural changes in the knee joint of the subjects

Next, we examined prevalence of PDFS MRI-detected OA structural changes of the whole knee joints (Table 2). Among OA structural changes of WORMS, osteophyte was most frequently observed (96.7%), consistent with the results of the previous study [21], followed by SBA (95.7%) and meniscus change (76.2%) (Table 2). When we focused on the medial compartment of the knee joint, the most frequent OA structural change was osteophyte (95.0%) (Table 2). SBA (93.4%) and meniscus change (61.7%) were also commonly observed (Table 2). The frequencies of the OA structural changes on the lateral compartment were less than those on the medial compartment of the knee joint, because the subjects with lateral type of knee OA (TFA <173) were excluded in our study.

**Table 2:** Prevalence of MRI-detected OA structural changes in the knee joint of the subjects.

MRI-detected OA changes	Incidence rate (%)		
	Total	Medial compartment	Lateral compartment
WORMS			
Cartilage	409 (35.7%)	374 (32.7%)	159 (13.9%)
BMA	383 (33.4%)	255 (22.2%)	151 (13.2%)
SBC	762 (66.6%)	507 (44.3%)	306 (26.7%)
SBA	1096 (95.7%)	1070 (93.4%)	852 (74.4%)
OP	1107 (96.7%)	1088 (95.0%)	1067 (93.2%)
Meniscus	873 (76.2%)	707 (61.7%)	574 (50.1%)
AME	–	1080 (94.3%)	–
ATO			
Bone part	–	1053 (92.0%)	–
Cartilage and bone parts	–	1140 (99.6%)	–

WORMS, whole-organ magnetic resonance imaging score; BMA, bone marrow abnormality; SBC, subchondral bone cyst; SBA, subchondral bone attrition; OP, osteophyte; AME, anterior meniscus extrusion; ATO, anterior tibial osteophyte.

AME was observed in 94.3% of the medial compartment on the sagittal section of PDSF MRI (Table 2). Analyses of ATO by PPDSF MRI demonstrated that prevalence of bone part width and full-length width of ATO is 92.0% and 99.6%, respectively (Table 2).

### 3.4 Association of MRI-detected OA structural changes with AME

We then examined whether the radiographic and MRI-detected OA structural changes are associated with AME (Table 3). Univariate analysis showed that both TFA (β ​= ​0.130, *p* ​< ​0.001) and medial JSW (β ​= ​−0.327, *p* ​< ​0.001) are correlated with AME. Among the OA structural changes evaluated by WORMS, cartilage lesion (β ​= ​0.233, *p* ​< ​0.001), BMA (β ​= ​0.309, *p* ​< ​0.001), SBC (β ​= ​0.225, *p* ​< ​0.001), osteophyte (β ​= ​0.118, *p* ​< ​0.001), and meniscus pathology (β ​= ​0.104, *p* ​= ​0.003) were correlated with AME. However, SBA was not correlated with AME (β ​= ​−0.038, *p* ​= ​0.397). Bone part width of ATO was also correlated with AME (β ​= ​0.764, *p* ​< ​0.001). When full-length width of ATO was evaluated, it was more closely correlated with AME (β ​= ​0.861, *p* ​< ​0.001). Multiple regression analysis indicated that among these MRI-detected OA structural changes, full-length width was most closely associated with AME (β ​= ​0.877; *p* ​< ​0.001) (Table 3), suggesting the strong association between AME and full-length width of ATO.

**Table 3:** Association between AME and MRI-detected OA structural changes in the medial compartment of the knee joint of the subjects.

OA changes		Univariable β	*p*	Multivariable β	*p*
Radiographic findings					
	TFA	0.130	<0.001	0.082	0.001
	Medial JSW	−0.327	<0.001	−0.198	0.002
MRI findings					
WORMS	Cartilage	0.233	<0.001	0.205	<0.001
	BMA	0.309	<0.001	0.221	<0.001
	SBC	0.225	<0.001	0.156	0.002
	SBA	−0.038	0.397	−0.058	0.196
	OP	0.118	<0.001	0.115	<0.001
	Meniscus	0.104	0.003	0.032	0.369
ATO width	Bone part	0.764	<0.001	0.763	<0.001
	Cartilage and bone parts	0.861	<0.001	0.877	<0.001

AME, anterior meniscus extrusion; TFA, tibiofemoral angle; JSW, joint space width; WORMS, whole-organ magnetic resonance imaging score; BMA, bone marrow abnormality; SBC, subchondral bone cyst; SBA, subchondral bone attrition; OP, osteophyte; ATO, anterior tibial osteophyte.

### 3.5 Detailed analysis of association between AME and ATO width

**Fig. 2:**
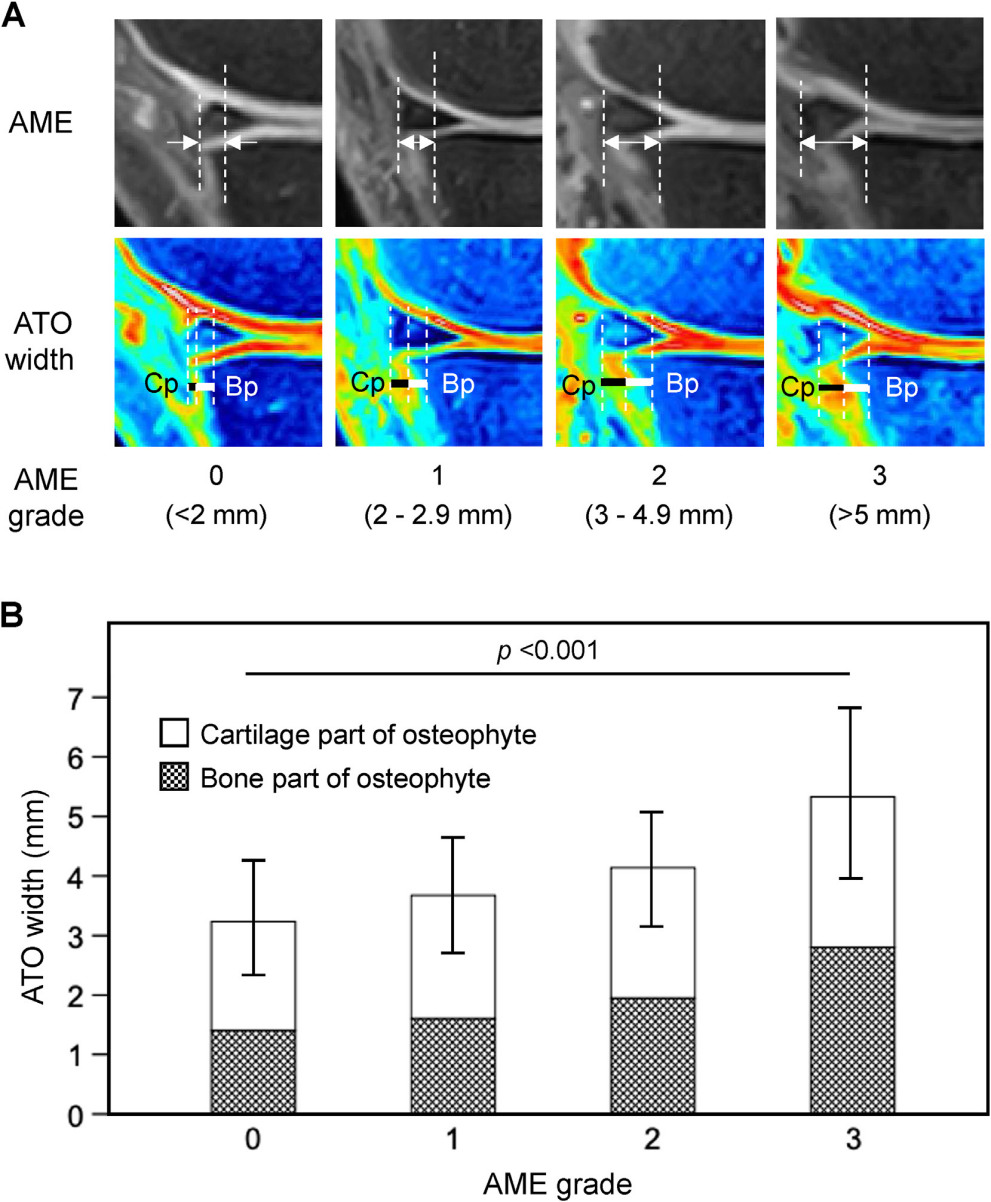
Association between AME and ATO width in the knee joints of representative subjects from our cohort. **(A)**, Representative sagittal PDFS and PPDFS MRI images of the medial tibio-femoral joints. The subjects were classified into four subgroups with AME grade 0 (<2 ​mm), grade 1 (2–2.9 ​mm), grade 2 (3–4.9 ​mm) or grade 3 (>5 ​mm). **(B)**, Correlation between ATO width and AME grade. ATO width was compared among the subgroups with AME grade 0, grade 1, grade 2 or grade 3. PDFS, proton density-weighted fat-suppressed; PPDFS, pseudo-colored PDFS; AME, anterior meniscus extrusion; ATO, anterior tibial osteophyte; Bp, bone part of osteophyte; Cp, cartilage part of osteophyte.

Association between AME and ATO width was analyzed by dividing the subjects into four subgroups with AME grade 0 (the width <2 ​mm), grade 1 (2–2.9 ​mm), grade 2 (3–4.9 ​mm) or grade 3 (≥5 ​mm) according to the degree of AME [19] (Fig. 2A). The ATO full-length width in each subgroup positively correlated with the AME grades (*p* ​< ​0.001) (Fig. 2B).

**Fig. 3:**
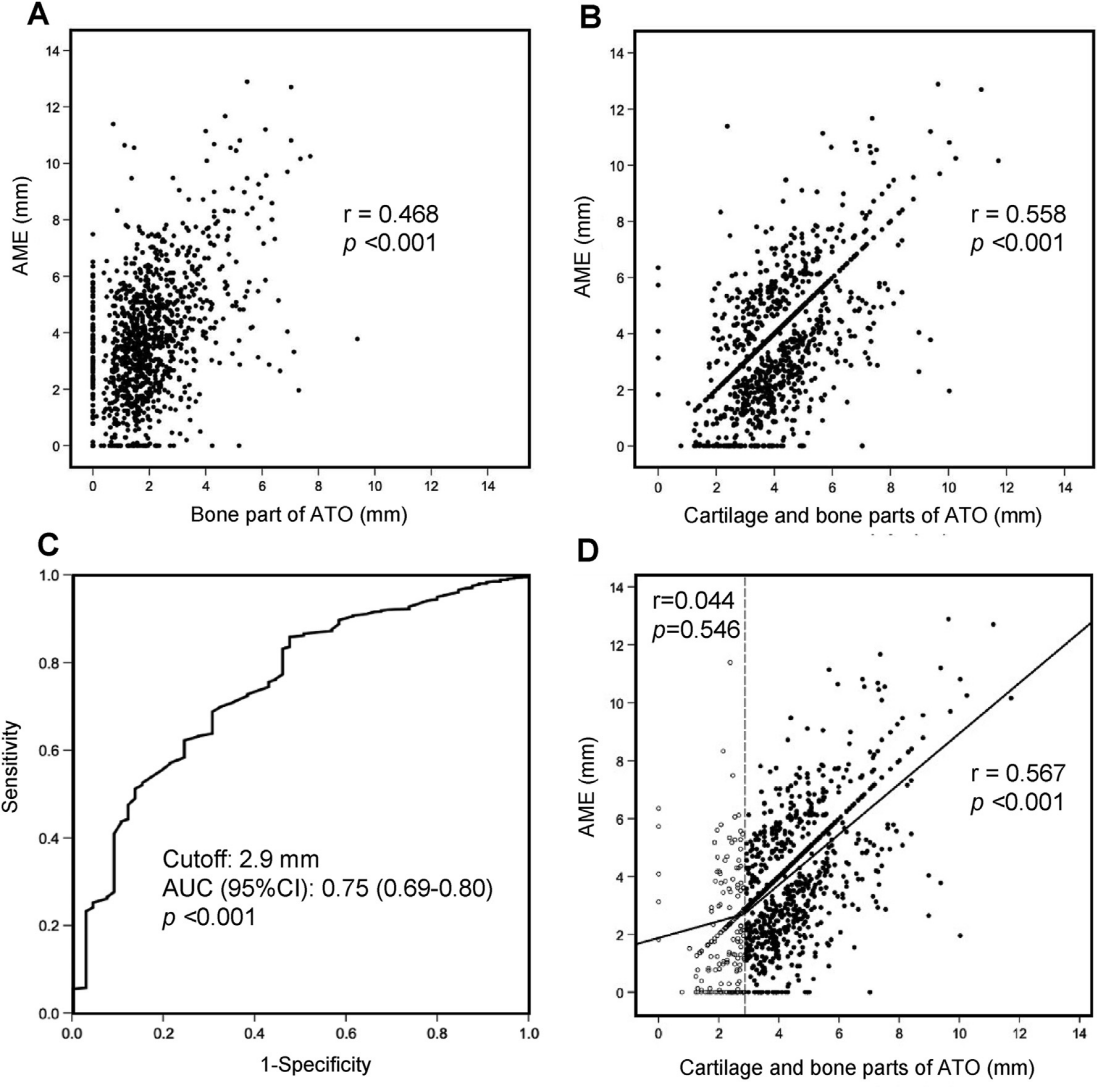
Correlations between AME and ATO width. **(A), (B),** Correlation between AME and bone part width of anterior tibial osteophyte (ATO) **(A)** and that between AME and full-length (cartilage and bone parts) width of ATO **(B)**. The correlations were examined by the Spearman's rank correlation analysis. AME, anterior meniscus extrusion; ATO, anterior tibial osteophyte; r, correlation coefficient. **(C)**, ROC curve for determining the presence of AME as an ATO width. **(D)**, Correlation between AME and ATO width among the subject subgroups divided into two groups according to the cut-off level of ATO (2.9 ​mm) for the presence of AME. AME, anterior meniscus extrusion; ATO, anterior tibial osteophyte; ROC, receiver operating characteristic (ROC) curve; AUC, area under the curve.

When bone part and cartilage part of ATO were separately evaluated, AME was significantly correlated with bone part width of ATO (r ​= ​0.468, *p* ​< ​0.001) (Fig. 3A). However, there were a certain number of subjects who showed AME without ATO (Fig. 3A). When full-length width of ATO was evaluated, AME was more strongly correlated with ATO (*r* ​= ​0.558, *p* ​< ​0.001) (Fig. 3B). In addition, there were a few subjects who showed AME (>0 ​mm) and ATO (0 ​mm), whereas there were a certain number of subjects who showed ATO (>0 ​mm) and AME (0 ​mm) (Fig. 3B).

### 3.6 Cut-off levels of ATO width for the presence of AME

Since there were 65 subjects who showed AME (0 ​mm) among 1145 subjects (5.7%), we calculated the AUC for the ROC curve. As shown in Fig. 3C, the AUC for the ROC curve for determining the presence of AME as evaluated by ATO width was 0.75 (95% confidence interval [CI], 0.69–0.80; *p* ​< ​0.001). The odds ratio for the presence of AME as evaluated by ATO width at 2.9 ​mm was 7.16 (95%CI, 4.23–12.15; *p* ​< ​0.001; adjusted by age, gender, BMI, and K-L) (Table 4).

**Table 4:** Risk for AME evaluated by ATO.

		ATO		OR (95%CI)	
		<2.9 ​mm, (*n* ​= ​188)	≥2.9 ​mm, (*n* ​= ​957)	Crude *p*	Adjusted *p*
AME	0 ​mm	35	30	6.83 (4.09–11.41) <0.001	7.16 (4.23–12.15) <0.001
	>0 ​mm	153	927		

*P* values were calculated by multinomial logistic regression after adjusting by age, sex, BMI, and K-L grade. AME, anterior meniscus extrusion; ATO, anterior tibial osteophyte; OR, odds ratio; K-L grade, Kellgren-Lawrence grade.

We also analyzed the correlation between ATO width and AME among the subject subgroups, and found that AME in the subgroup who had ≥2.9 ​mm of ATO width was significantly correlated with ATO width (*r* ​= ​0.567, *p* ​< ​0.001), whereas no significant correlation was observed in the subjects who had <2.9 ​mm of ATO width (*r* ​= ​0.044, *p* ​= ​0.546) (Fig. 3D).

## 4 Discussion

In the present study, to the best of our knowledge, we have demonstrated for the first time that AME and ATO are frequently observed in elderly populations of the large-scale cohort. In addition, our data have indicated that AME is most closely associated with ATO width among the MRI-detected OA structural changes of the knee joint. These data suggest a possible relationship between development of AME and ATO formation.

Recent clinical studies using MRI have revealed a critical role of meniscus pathologies such as meniscus tear and extrusion in the initiation of articular cartilage destruction in knee OA [3,16,[30], [31], [32]]. Incidence of meniscus tear is reported to be age-dependently increased [33]. In accord with these reports, previous study has demonstrated that meniscal damage is prevalent among elderly individuals with knee pain, especially in those with knee OA [34]. In addition, our preliminary and parallel studies on the elderly subjects of our cohort (the BHS) have disclosed that nearly half and >70% of the subjects have medial meniscus tear and MME, respectively [23,35,36]. However, to the best of our knowledge, no data have been reported about the prevalence of AME in elderly populations. The present study therefore provides the first evidence that AME is developed in most of the elderlies (94.3% of the subjects).

Since cartilage tissue is undetectable by radiography and conventional PDFS MRI, incidence and size of osteophytes in knee OA joint have been underestimated. To overcome this problem, we used T2 mapping MRI for evaluation of osteophytes in early-stage knee OA patients and demonstrated the usefulness of T2 mapping MRI to determine full-length (both cartilage and bone parts) width of osteophytes [7]. However, T2 mapping MRI is a time-consuming method and inapplicable for large-scale population-based cohort study. Therefore, development of a new method of PPDFS imaging and its application to our cohort were essential to measurement of full-length width of ATO and analyses of the relationship between AME and ATO width. Our preliminary and parallel studies on the analysis of osteophytes in the cohort study showed that the PPDFS images are comparable to T2 mapping, providing even higher contrast resolution than T2 mapping images and that this method is applicable for evaluation of the osteophytes in cohort study [23]. In the present study, both cartilage and bone parts of ATO were detected by PPDSF images of the elderlies and ratios of the cartilage part to the bone part appeared to be similar (Fig. 2B), resulting in almost double in size for full-length width of ATO compared to the bone-part width of ATO.

Osteophyte was thought as a secondary response to repair damage of the structure in OA knee joint [37]. However, accumulated lines of evidence have shown that osteophyte is the most common structural change of the OA knee joint, frequently preceding cartilage damage [21]. Osteophyte is known to develop in the short term under some special conditions. We have recently reported that patients who had anterior cruciate ligament injury and underwent ligament reconstruction surgery develop osteophytes at ∼7 months on average after the surgery [38]. In addition, meniscal tear is known to be the strongest structural risk factor for development of tibiofemoral osteophytes in the middle-aged subjects with K-L grade 0 [39], suggesting the involvement of local biomechanical factors in early osteophyte formation. However, there are many unsettled questions about the mechanism of osteophyte formation [10,40,41], and further studies are definitely needed to better understand the developmental process and risk factors of ATO formation.

**Fig. 4:**
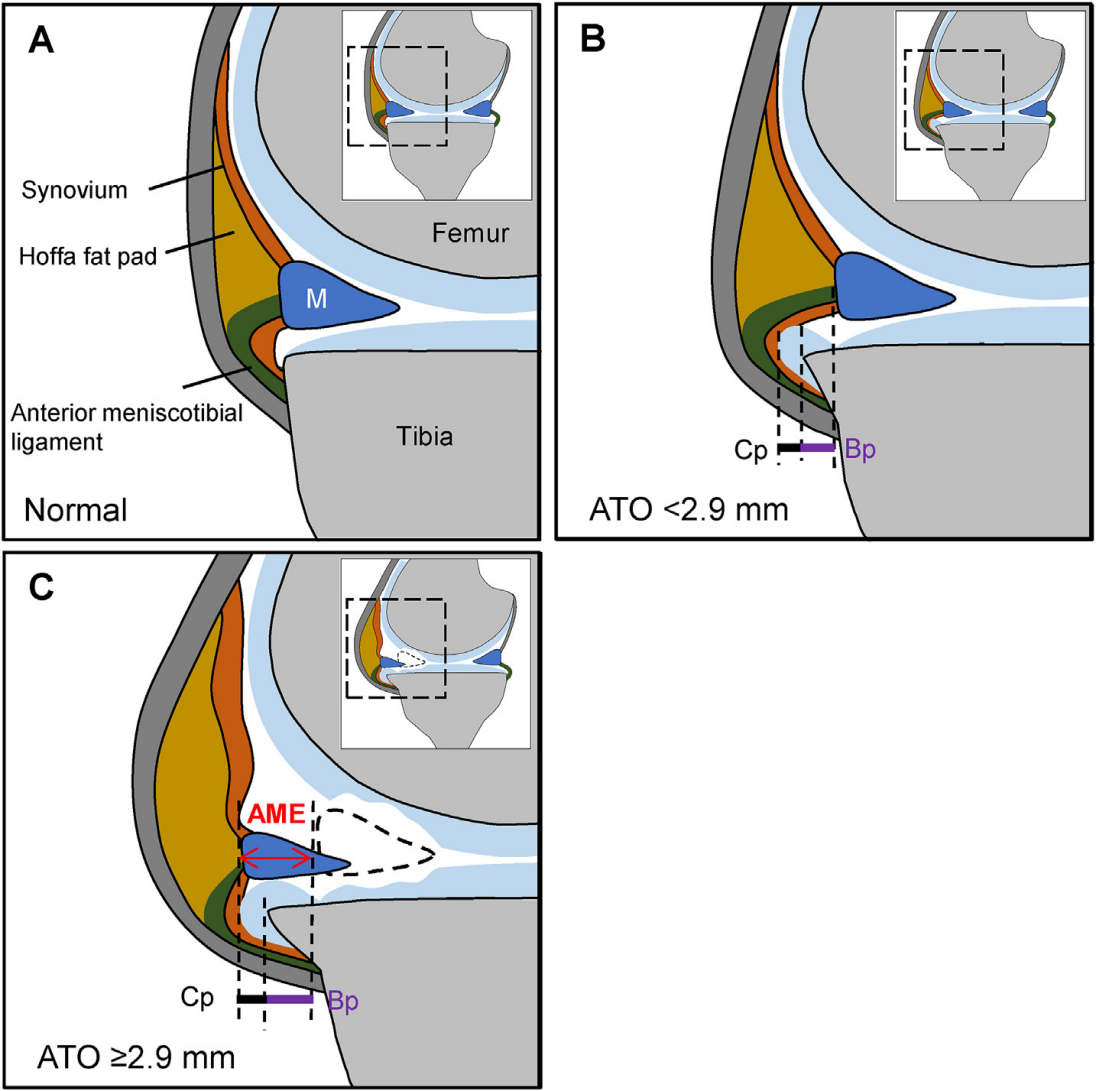
Schematic illustrations showing the hypothesis on relationship between anterior tibial osteophyte (ATO) and anterior meniscus extrusion (AME) in OA knee joint. **(A)**, Normal knee joint on sagittal image. The anterior region of the medial meniscus is attached to the anterior tibial condyle by the anterior menisco-tibial ligament, and the medial part of the Hoffa fat pad is located on the patellar ligaments. **(B)**, Sagittal image of OA knee joint with small ATO (width ​< ​2.9 ​mm) measured on PPDFS images. Since the ATO may be settled in the space present between the anterior menisco-tibial ligament and the anterior tibial edge, neither shift of the meniscus nor development of AME occurs. **(C)**, Sagittal image of OA knee joint showing relationship between AME and larger ATO (width ​≥ ​2.9 ​mm) measured on PPDFS images. The ATO may press the anterior menisco-tibial ligament and displace the anterior region of the meniscus, leading to AME. Bp, bone part of osteophyte; Cp, cartilage part of osteophyte; M, medial meniscus; PPDFS, pseudo-colored proton density-weighted fat-suppressed.

One of the intriguing findings in the present study is that full-length width of ATO is directly correlated with AME. According to our findings obtained by the PPDFS images and anatomical characteristics of the knee joint [5,6], we propose the following hypothesis on the relationship between ATO and AME (Fig. 4). Under normal conditions, the medial meniscus is attached to the medial tibial condyle by the menisco-tibial ligament (coronary ligament) [6]. However, in contrast to tight linkage of the medial region of the meniscus to the tibial condyle by the ligament [6,7], the anterior region of the medial meniscus seems to be more loosely attached to the anterior tibial condyle by the anterior menisco-tibial ligament, and the medial part of the Hoffa fat pad is located on the medial patellar ligaments [6,42] (Fig. 4A). Therefore, when width of ATO is small, i.e., less than 2.9 ​mm in the present study, the edge of the anterior region of the medial meniscus may stay within the normal position of the meniscus without a shift to anterior site (Fig. 4B). However, once ATO becomes larger and exceeds the width more than 2.9 ​mm, AME may be induced by the mechanical pressure of the anterior menisco-tibial ligament by ATO, showing that the length of AME is almost similar to the full-length width of ATO (Fig. 4C).

In our preliminary study on a small number of the healthy subjects less than 60 years of age who had no radiographic knee OA (K-L grade 0), we found that AME and ATO are detected in only less than 20% of the group of 20–39 years of age, showing neither AME nor ATO in the subjects of 20–29 years of age, but incidence rate of AME and ATO is increased to about 70% in the subjects of 40–59 years of age (Arepati et al. unpublished data). Similar high prevalence of osteophyte in middle aged and elderly people is reported by the MRI study on a cohort (Framingham Osteoarthritis Study) [21]. They showed that osteophyte is detected in 68% and 78% of the subjects aged ≥50 to <60 and ≥60 to <70, respectively, reaching 80% in the subjects aged ≥80. Thus, our preliminary findings together with the data in the present and previous studies indicate that AME and ATO are pathological event with aging in healthy subjects and suggest their possible involvement in the initiation and progression of knee OA. To prove our hypothesis, however, further studies such as longitudinal analyses of prevalence of ATO and AME and their relationship in early-stage knee OA patients, those in larger numbers of healthy subjects with different ages, and/or studies in experimental knee OA models are necessary.

Recent clinical studies using MRI or ultrasonography have demonstrated that synovitis is commonly observed in OA knee joints [43]. By non-contrast-enhanced MRI, effusion-synovitis on axial image and Hoffa-synovitis on sagittal image are the surrogates to identify OA synovial inflammation and both are reportedly a strong risk factor (a precursor) for radiographic knee OA [44]. Although OA synovitis was previously thought to be a consequence resulting from macrophage phagocytosis of cartilage debris [45], growing evidence has suggested that many OA risk factors such as aging, obesity, mechanical loading, cytokines, metabolites and crystals are implicated for induction and/or stimulation of synovitis in OA [46]. The molecular mechanism by which Hoffa-synovitis is induced remains unknown at the present time. However, when ATO grows large enough to press the anterior menisco-tibial ligament, aberrant excessive mechanical loading generated repeatedly during joint movement may activate Hoffa fat pad cells and synovial cells, leading to induction and synthesis of inflammatory mediators such as interleukins, cyclooxygenase 2 and nitric oxide synthase 2 [46]. Thus, it is possible to speculate that ATO may play a dual role in induction of Hoffa-synovitis and AME, both of which are reportedly associated with knee pain [47].

The present study has some potential limitations. First, we used the PPDFS method by pseudo-colorization of PDFS images [23,38]. Accuracy of this method was confirmed by comparison of the PPDFS images with T2 mapping MRI images and histology of the surgically removed knee joint tissues obtained from patients with symptomatic knee OA, but not normal subjects [23]. Therefore, it is not completely guaranteed whether this method is sufficient to evaluate subtle changes in knee joints including osteophytes in elderlies who do not require medical treatment for knee OA. Accuracy of the method should be further assessed by analyses of the knee joints of human subjects including cadavers. However, because of time reduction and efficiency for evaluation of numerous samples, the method was essential for analyses of the samples in the present cohort study. Second, in the present study, the same observers measured both AME and ATO width. Thus, there is a risk of confirming our hypothesis, and this must be one of the biases of the present study. Third, our cohort included only the subjects living in an urban part of Japan. Therefore, generalization for other races from the results may have some bias. In addition, the data obtained in the present study may not be applicable for elderly people living in other areas such as mountainous or coastal areas.

In conclusion, we have demonstrated that AME and ATO are observed in 94.3% and 99.6% of the elderly populations (1080/1145 subjects and 1.140/1145 subjects, respectively), and that AME is most closely associated with full-length (cartilage and bone parts of osteophytes) width of ATO (β ​= ​0.877, *p* ​< ​0.001; multiple regression analysis) among the MRI-detected OA structural changes. Our study provides the first evidence on the close relationship between AME and ATO in the knee joint.

## Funding

This study was supported in part by Grant-in-Aid for Scientific Research from the 10.13039/501100000646Japanese Society for the Promotion of Science (JSPS) to MI (15K10494 and 18K09082), HK (15K20019 and 18K09083), SH (16K20069), TA (19K17209) and YO (16H05454 and 19H03788). This study was also funded in part by a JOA (10.13039/100016626Japanese Orthopaedic Association)-Subsidized Science Project Research 2022–2024, by a High Technology Research Center Grant and the Program for the Strategic Research Foundation at Private Universities (2014–2019) from the Ministry of Education, Culture, Sports, Science and Technology of Japan (MEXT), and by a Center of Innovation (COI) program, one of the main funding programs under the Center of Innovation Science and Technology based Radical Innovation and Entrepreneurship Program (COI STREAM), which was launched in 2013 by the 10.13039/501100001700MEXT and operated by 10.13039/501100002241Japan Science and Technology Agency (JST), to 10.13039/501100005731Juntendo University.

## Author contribution

AA, YN, HK, YS, YT, and MI conceived and designed the study. AA, YN, TA, LL, HA, MM, SW, and JS collected and registered data of the subjects. AA, YN, HK, TA, LL, SH, MK, SA, HW, RK, TNK, YO, and MI had the major role in analysis and interpretation of the data, and contributed to drafting the report. SH, HK-LL, YO and MI also supervised the statistical analysis. All authors have read and approved the final manuscript.

## Availability of data and materials

The datasets used and/or analyzed during the current study are available from the corresponding authors on reasonable request.

## Ethics approval and consent to participate

This cross-sectional study was approved by the Hospital Ethics Committee of Juntendo University Hospital (approval numbers: 2015078, 2016138, 2016131, 2017121, and 2019085).

## Consent for publication

Not applicable.

## Declaration of competing interest

The authors declare that they have no known competing financial interests or personal relationships that could have appeared to influence the work reported in this paper.

## Appendix A Supplementary data

Supplementary Fig. S1 Representative data of PDFS and PPDFS MRI images to measure AME and ATO width in normal subjects without or with osteophyte. **(A)**, The sagittal PDFS and PPDFS images of normal subject without osteophyte. Note that anterior edge of the tibia is almost rectangular in shape. Vertical dotted line indicates the outer edge of the medial meniscus and the border of the tibia, both of which are almost identical in this case. **(B)**, Measurement of AME and ATO width on the sagittal PDFS and PPDFS images. AME and ATO width were measured as the distance between the vertical dotted line drawn at the outer edge of the medial meniscus and the vertical dotted line drawn at border of the tibia, making the rectangle-shaped anterior edge of the tibia appear. PPDFS MRI view was prepared by pseudo-coloring the PDFS MRI image as described in the Methods. Vertical dotted lines indicate the anterior edge of the tibia excluding osteophyte, the boundary between the bone part of osteophyte (Bp) (white horizontal line) and cartilage part of osteophyte (Cp) (black horizontal line), and the outer edge of the cartilage part of osteophyte. White horizontal line with arrows indicates full-length (Bp plus Cp) width of ATO. PDFS, proton density-weighted fat-suppressed; PPDFS, pseudo-colored PDFS; M, medial meniscus; AME, anterior meniscus extrusion; ATO, anterior tibial osteophyte. Supplementary Fig. S1
