# Supplementary figures and images for: Anterior meniscus extrusion is associated with anterior tibial osteophyte width in knee osteoarthritis – The Bunkyo Health Study
Source: Osteoarthr Cartil Open. 2023 Apr 20;5(3):100364. doi: 10.1016/j.ocarto.2023.100364 (PMC10189494; doi:10.1016/j.ocarto.2023.100364)

## Slide 1
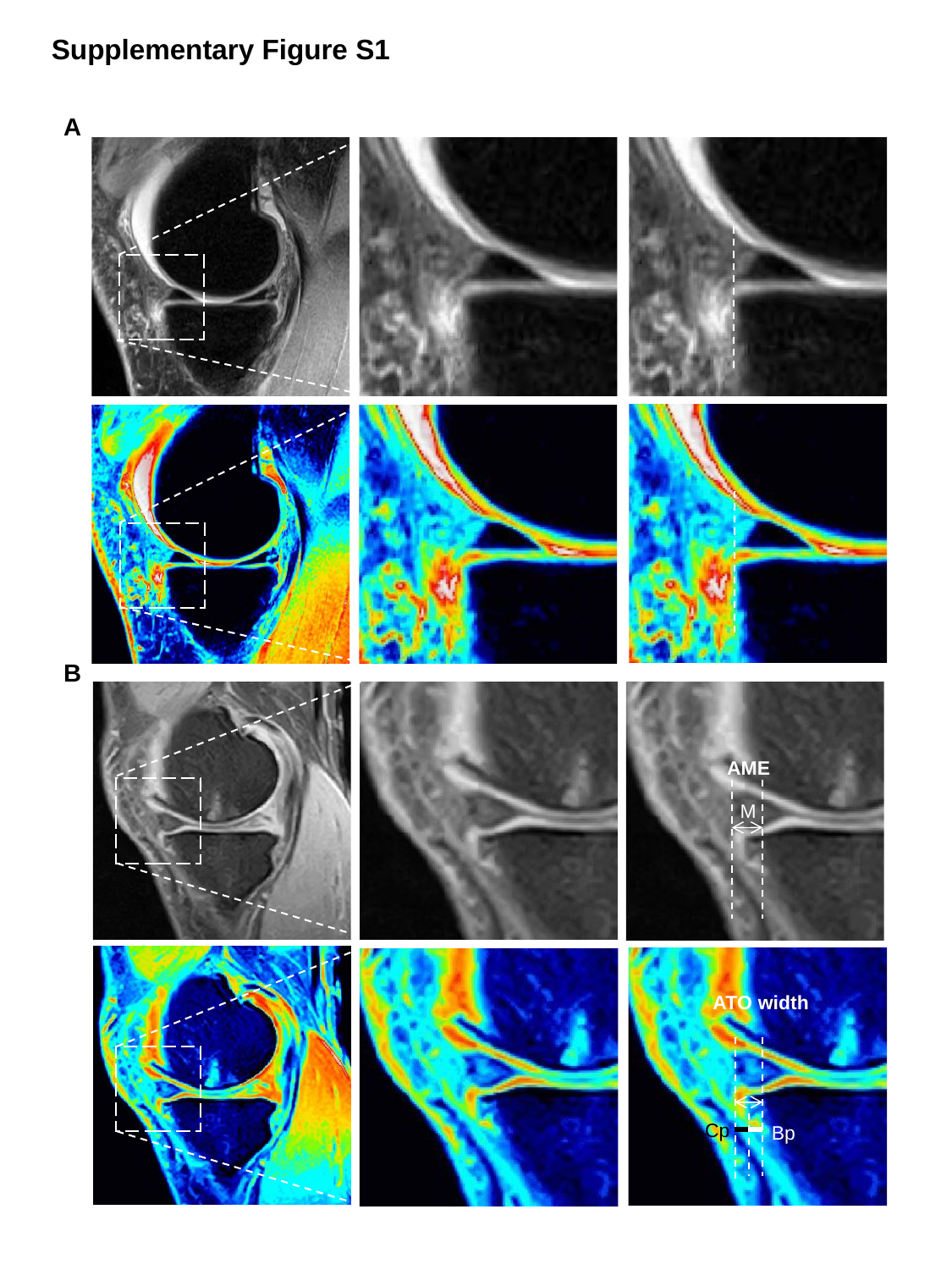

Supplementary Figure S1
A
B
AME
M
ATO width
Cp
Bp

Supplement: Supplementary Fig. S1 — Representative data of PDFS and PPDFS MRI images to measure AME and ATO width in normal subjects without or with osteophyte. (A), The sagittal PDFS and PPDFS images of normal subject without osteophyte. Note that anterior edge of the tibia is almost rectangular in shape. Vertical dotted line indicates the outer edge of the medial meniscus and the border of the tibia, both of which are almost identical in this case. (B), Measurement of AME and ATO width on the sagittal PDFS and PPDFS images. AME and ATO width were measured as the distance between the vertical dotted line drawn at the outer edge of the medial meniscus and the vertical dotted line drawn at border of the tibia, making the rectangle-shaped anterior edge of the tibia appear. PPDFS MRI view was prepared by pseudo-coloring the PDFS MRI image as described in the Methods. Vertical dotted lines indicate the anterior edge of the tibia excluding osteophyte, the boundary between the bone part of osteophyte (Bp) (white horizontal line) and cartilage part of osteophyte (Cp) (black horizontal line), and the outer edge of the cartilage part of osteophyte. White horizontal line with arrows indicates full-length (Bp plus Cp) width of ATO. PDFS, proton density-weighted fat-suppressed; PPDFS, pseudo-colored PDFS; M, medial meniscus; AME, anterior meniscus extrusion; ATO, anterior tibial osteophyte. [file mmc1.pptx]
